# Recent Advances in the Genetic Transformation of Coffee

**DOI:** 10.1155/2012/580857

**Published:** 2012-08-29

**Authors:** M. K. Mishra, A. Slater

**Affiliations:** ^1^Central Coffee Research Institute, Coffee Research Station, Chikmagalur, Karnataka 577117, India; ^2^The Biomolecular Technology Group, Faculty of Health and Life Sciences, De Montfort University, Gateway, Leicester LE1 9BH, UK

## Abstract

Coffee is one of the most important plantation crops, grown in about 80 countries across the world. The genus *Coffea* comprises approximately 100 species of which only two species, that is, *Coffea arabica* (commonly known as arabica coffee) and *Coffea canephora* (known as robusta coffee), are commercially cultivated. Genetic improvement of coffee through traditional breeding is slow due to the perennial nature of the plant. Genetic transformation has tremendous potential in developing improved coffee varieties with desired agronomic traits, which are otherwise difficult to achieve through traditional breeding. During the last twenty years, significant progress has been made in coffee biotechnology, particularly in the area of transgenic technology. This paper provides a detailed account of the advances made in the genetic transformation of coffee and their potential applications.

## 1. Introduction

Coffee is one of the most important agricultural commodities, ranking second in international trade after crude oil. The total global production of green coffee is above 134.16 million bags (60 kg capacity) with a retail sales value in excess of $22.7 billion during 2010-11 in the world market [1]. Coffee is grown in about 10.2 million hectares land spanning over 80 countries in the tropical and subtropical regions of the world especially in Africa, Asia, and Latin America. The economics of many coffee growing countries depends heavily on the earnings from this crop. More than 100 million people in the coffee growing areas worldwide derive their income directly or indirectly from the produce of this crop.

Coffee trees belong to the genus *Coffea* in the family Rubiaceae. The genus *Coffea* L. comprises more than 100 species [2], of which only two species, that is, *C. arabica* (arabica coffee) and *C. canephora* (robusta coffee), are commercially cultivated. Another coffee species, *Coffea liberica* is also cultivated in a small scale to satisfy local consumption. Almost all the coffee species are diploid (2n = 2x = 22) and generally self-incompatible except *C. arabica* which is a natural allotetraploid (2n = 4x = 44) self-fertile species [3]. In the consumer market, *C. arabica* is preferred for its beverage quality, aromatic characteristics, and low-caffeine content compared to robusta, which is characterized by a stronger bitterness, and higher-caffeine content. Arabica contributes towards 65% of global coffee production [4].

*C. arabica* is mainly native to the highlands of Southwestern Ethiopia with additional populations in South Sudan (Boma Plateau) and North Kenya (Mount Marsabit) [5–8]. The *C. arabica* varieties grown all over the world are derived from either the “Typica” or “Bourbon” genetic base, which has resulted in low-genetic diversity among cultivated arabicas. In contrast, *C. canephora* has a wide geographic distribution, extending from the western to central tropical and subtropical regions of the African continent, from Guinea and Liberia to Sudan and Uganda with high genetic diversity in the Democratic Republic of Congo [9]. *C. canephora* maintains heterozygosity due to its cross-pollinating nature.

## 2. Coffee Breeding and Its Limitations

Coffee breeding is largely restricted to the two species, *C. arabica* and *C. canephora,* that dominate world coffee production. However, *C. liberica* and *C. congensis *have contributed useful characters to the gene pool of *C. arabica* and *C. canephora, *respectively, through natural and artificial interspecific hybridisation. In *C. arabica*, initial breeding objectives were to increase productivity and adaptability to local conditions. To achieve these objectives, breeding strategies were directed towards identification of superior plants in the population and their propagation and crossing with existing cultivars. These early breeding efforts, which were carried out from 1920 to 1940, had considerable success in identifying and developing vigorous and productive cultivars. Several of these varieties such as Kents and S.288 from India, Mundo Novo, Caturra and Catuai from Brazil, and Blue Mountain from Jamaica, are still under commercial cultivation. These cultivars are suggested to have a larger degree of genetic variability than the base population [10]. The appearance of coffee leaf rust (*Hemileia vastatrix *Berk and Br) in epidemic scale in Southeast Asia between 1870 and 1900 had a devastating effect on arabica coffee cultivation in several coffee growing countries. This has changed the breeding focus worldwide with emphasis now given to disease resistance. This has resulted in the introduction of other tolerant species, especially *C. canephora,* in many countries. Until now, *C. canephora* has provided the major source of disease and pest resistance traits such as coffee leaf rust (*H. vastatrix*), coffee berry disease (*Colletotrichum kahawae*), and root-knot nematode (*Meloidogyne* spp.) not available in *C. arabica*. Besides, *C. canephora*, other diploid species such as *C. liberica* has been used as source of resistance to leaf rust [11] and *C. racemosa* for imparting resistance to coffee leaf minor [12]. Further, the cultivation of *C. arabica *with other diploid species such as *C. canephora* and *C. liberica *in close proximity has resulted in spontaneous hybrids in many countries. Natural interspecific hybrids such as Hybrido-de Timor (a hybrid between *C. arabica* and *C. canephora* [13] from Timor island), Devamachy (a hybrid between *C. arabica* and *C. canephora*),and S.26 (a hybrid between *C. arabica* and *C. liberica, which* both originated in India [14]) are the main source of resistance to pest and disease and extensively used in *C. arabica* breeding programmes.

Like *C. arabica*, improvement of *C. canephora* was originally aimed at increasing productivity, and improving bean size and liquor quality. The breeding methods adopted for *C. canephora* involvedmass selection and intra- as well as interspecific hybridization. Varieties like Apoata of Brazil, S.274 of India, and Nemaya of Central America were derived through mass selection. The spontaneous diploid interspecific hybrid between *C. canephora var. ugandae* and the *C. congensis *(called Congusta in Indonesia), and the C × R hybrid variety developed through artificial hybridisation between *C. congensis* and *C. canephora* in India are examples of improved robusta cultivars developed through interspecific hybridisation.

Although conventional breeding is mainly used for coffee improvement, it is a long process involving several different techniques, namely selection, hybridization, and progeny evaluation. A minimum of 30 years is required to develop a new cultivar using any of these methods. Further, the long generation time of the coffee tree, the high cost of field trials, the lack of accuracy of the breeding process, the differences in ploidy level between *C. arabica* and other diploid species, and the incompatibility are all major limitations associated with conventional coffee breeding. In addition to these, genetic resistance to coffee white stem borer (*Xylotrechus quadripes*) and coffee berry borer (*Hypothenamus hampei*), drought and cold tolerance, and herbicide resistance are some of the features that are not easily available in the coffee gene pool or are difficult to incorporate using conventional breeding. Another constraint that hinders the arabica coffee improvement programme is the selection of genetically diverse parental lines for hybridization and the identification of hybrids at an early stage of plant growth based on morphological traits. This is because most of the commercial arabica cultivars are morphologically identical and not easily distinguishable from one another. Uniformity of morphological traits in *C. arabica* could be attributed to the origin of the species, its narrow genetic base and self-fertile nature. In coffee, identification of cultivars is mainly based upon phenotypic features, but this approach is not reliable and is subject to environmental influences, mainly because of the long generation time of the coffee trees. In some countries of Asia, Latin America, and Africa, coffee is cultivated under shade in varied agroclimatic conditions and displays remarkably different morphologies in various microclimatic zones. In view of the above, it becomes imperative to develop alternative techniques that are reliable, quick, and efficient for discriminating between coffee cultivars. Among the various markers available for genetic analysis in coffee, molecular markers are more efficient, precise, and reliable than other markers for discriminating closely related species and cultivars. The DNA-based markers have the potential of complimenting coffee breeding and improvement program in form of marker-assisted selection (MAS).

## 3. Molecular Markers and Coffee Genetic Improvement

Various molecular markers, such as restriction fragment length polymorphism (RFLP), random amplified polymorphic DNA (RAPD), amplified fragment length polymorphism (AFLP), intersimple sequence repeat (ISSR), simple sequence repeats (SSR), and expressed sequence tag derived simple sequence repeats (EST-SSR) have been used in coffee genetic diversity studies [46–51]. In addition to the above, a large number of commercial coffee samples of American, Indian, and African origin were also analyzed using highly polymorphic SSR markers which revealed that Indian cultivars were genetically diverse from the American and African cultivars [52]. More recently, a new type of molecular marker known as a sequence related amplified polymorphism (SRAP) was used in genetic diversity analysis of coffee cultivars and species [53, 54]. SRAP markers were also successfully used to discriminate between parents in hybrid identification [55, 56] and therefore has great potential in coffee breeding programmes. In addition to the above, single-nucleotide polymorphisms (SNPs) and PCR-RFLP markers were used in coffee genome analysis, which revealed that in *C. arabica*, polymorphisms are created by paralogous chromosomes, whereas homozygosity of many genes is maintained by the self-fertile nature of the species [57]. These results further demonstrated that in allopolyploid *C. arabica,* the two parental genomes remain separated and exhibit multiple allelic inheritance patterns, and these findings will be very important for designing strategies and decisions in breeding programmes as well as in sequencing projects. In recent years, concerted efforts have been made by several laboratories across the world, under the International Coffee Genome Network (ICGN) programme, to sequence the coffee genome by using high-throughput sequencing technology which will unravel several key aspects of the coffee genome that may be useful for coffee genetic improvement. In addition to molecular markers, a two-dimensional protein mapping technique was also used to differentiate green coffee samples [58]. A detailed review of the role of various molecular markers in coffee is already available [59] and therefore beyond the scope of the present review.

## 4. The Need for Genetic Transformation of Coffee

Since its initial application to plants more than 25 years ago, genetic transformation has become an indispensable tool in plant molecular biology and functional genomics research [60]. Genetic transformation technology is considered as an extension of conventional plant breeding technologies [61]. It offers unique breeding opportunities by introducing novel genetic material irrespective of the species barrier and creating phenotypes with desired traits that are not available in the germplasm pool of crop plants. The major objectives for using genetic engineering technique in coffee are to introduce new traits in to elite coffee genotypes, develop new cultivars with desirable traits such as pest and disease resistance, herbicide resistance, drought and frost tolerance, and improved cup quality, which are not possible to incorporate using traditional breeding techniques. The recent developments in coffee transcriptomics and the availability of large amounts of expressed sequence tag (EST) data from both *C. canephora* and *C. arabica* [62–64], as well as the development of coffee bacterial artificial chromosome (BAC) genomic libraries [65, 66], have opened up new possibilities in the area of coffee functional genomics. A key component of most functional genomics approaches is the availability of a highly efficient transformation system useful for designing strategies for gene identification, elucidation of gene functions, regulation and interaction of genes and gene expression analysis to understand the involvement of genes, in coffee biological processes. This will help in precisely targeting the trait of interest using various transformation tools (genes and promoters) with increase probability of success in reducing economic costs.

Genetically modified coffee plants have been produced by different research groups in the world [21, 25, 26, 29, 34, 37, 67]. Despite significant advances over the last 20 years, coffee transformation is far from a routine procedure in many laboratories [35]. The objective of this paper is to provide an update on coffee genetic transformation over the last decade, including the *in vitro* methods used for plant generation.

## 5. *In Vitro* Plant Regeneration

The establishment of an efficient regeneration system is important for genetic transformation of coffee. Various *in vitro* multiplication methods such as somatic embryogenesis, meristem and axillary bud culture, and induction of adventitious buds have been reported using different types of tissue in various coffee species [68, 69].

### 5.1. Somatic Embryogenesis

The initiation and development of embryos from somatic tissues without the involvement of sexual fusion are known as somatic embryogenesis. In coffee, induction of somatic embryogenesis and plant regeneration was first reported from the internodal explants of *C. canephora* [70]. In *C. arabica, *calluses were successfully induced from seeds, leaves, and anthers of two different cultivars, that is, Mundo Novo and Bourbon Amarelo [71]. During the last 35 years, a number of protocols for somatic embryogenesis have been developed for various genotypes of coffee [68]. The first protocol to obtain calli with high embryogenic potential from the leaf explants of *C. arabica* cv. Bourbon used two different culture media compositions: a first “conditioning” medium and a second “induction” medium [72, 73]. The availability of auxins is critical for the induction of embryogenic calli [72]. In coffee, both high-frequency somatic embryogenesis (HFSE) and low-frequency somatic embryogenesis (LFSE) were established. 2,4-D strongly increases HFSE in combination in primary cultures where as IBA and NAA combined with K increase LFSE. During somatic embryo induction in *C. arabica* cv. Caturra Rojo, two types of cell clusters, embryogenic and nonembryogenic were observed [74]. The differences in gene expression at both RNA and protein levels were observed between the embryogenic and nonembryogenic cell clusters. Further, it was observed that the number of genes turned off in somatic cells to allow for the change from somatic to embryogenic state is higher than those genes that are turned on [74].

In coffee, somatic embryogenesis follows two distinct developmental patterns: (1) direct somatic embryogenesis, where embryos originate directly from the explants and (2) indirect somatic embryogenesis, where embryos are derived from an embryogenic dedifferentiated tissue (callus). However, both direct and indirect somatic embryos of coffee formed from leaf segments and callus, respectively, have a unicellular origin [75]. Various attempts were made to reduce the time needed for embryogenesis and increase the embryogenesis frequency in coffee. Triacontanol, silver nitrate (AgNO_3_), salicylic acid, thidiazuron, and 6-(3-methyl-3-butenylamino) purine (2ip) are the widely used growth regulators in coffee embryogenesis. Interestingly, picomolar concentrations of salicylates reported to induce cellular growth and enhance somatic embryogenesis in *C. arabica* tissue culture [76]. Similarly, triacontanol, as well as silver nitrate, at low concentration in combination with indole-3-acetic acid (IAA) and benzyladenine (BA) induced direct somatic embryogenesis in both species of *C. arabica* and *C. canephora* [77, 78]. Additionally, thidiazuron (TDZ) also induced direct somatic embryos from the cultured leaf explants of *C. canephora* cv. C × R [79]. In *C. canephora*, the embryogenic response of the explants has been shown to increase by the addition of polyamines, either alone or in combination with silver nitrate. It has been observed that incorporation in the *in vitro *culture medium of inhibitors of the polyamine biosynthetic pathway such as D,L-.alpha-difloromethylornithine (DFMO) and D,L-.alpha-difloromethylarginine (DFMA) significantly reduced the embryogenic response of the explants in *C. canephora,* indicating the pivotal role played by polyamines in coffee somatic embryogenesis [80]. Besides the polyamines, indoleamines (melatonin and serotonin) as well as calcium and calcium ionophores (A23187) have also been shown to be beneficial in inducing somatic embryogenesis [81]. Apart from exogenous growth hormones, ethylene and dissolved oxygen concentration play a crucial role in coffee somatic embryogenesis [82, 83].

The use of somatic embryos on an industrial scale was achieved by inducing somatic embryos of *C. arabica* in liquid medium using bioreactors [84, 85]. The yield of embryos achieved was about 46,000 embryos/3L Erlenmeyer flask (after 7 weeks of culture). Various other workers also reported the production of somatic embryos for industrial use [86, 87]. Extensive studies were carried out in the use of conventional and temporary immersion system for coffee somatic embryo production [68, 88, 89]. However, to date the major obstacle associated with production of somatic embryos on a commercial scale is synchronisation of embryogenesis and conversion of plantlets.

### 5.2. Micropropagation

The coffee plant has a single apical meristem with each axil leaf having 4-5 dormant orthotropic buds and two plagiotropic buds. The plagiotropic buds only start development from the 10th to 11th node. For apical meristem culture and the culture of dormant buds, both orthotropic and plagiotropic buds were cultured for obtaining plantlets. Microcuttings or nodal culture comprise a tissue culture approach which involves culturing nodal stem segments carrying dormant auxiliary buds and stimulating them to develop. Each single segment can provide 7–9 microcuttings every eighty days. Most of these studies were carried out during the 1980s, and these topics have been reported in an earlier review [68].

Several studies have been carried out with a view to micropropagating superior coffee genotypes using apical or axillary meristem culture and nodal culture [68]. A maximum of nine shoots was obtained per one shoot explant [90]. Culture of microcuttings in a temporary immersion system resulted in a 6-fold increase in the multiplication rate, in comparison with microcuttings multiplied on solid medium [91, 92]. The field performance of embryo-generated plants was reported and showed a normal response in terms of physiology and yield. The genetic fidelity of micropropagated plants of *C. canephora* obtained through somatic embryogenesis was assessed in a large-scale field trial [93]. A total number of 5067 trees regenerated from five to 7-month-old embryogenic cell suspension cultures were planted in the Philippines and in Thailand for comparison with control plants derived from auxiliary budding *in vitro*. No significant differences in yield and morphological features were observed between the somatic seedlings and microcutting derived trees [93]. However, in contrast to the above, several studies have clearly demonstrated culture-induced variation and regeneration of somaclonal variants in coffee obtained through direct and indirect somatic embryogenesis [94–96]. Detailed molecular analysis of the plantlets of *C. arabica* derived from high-frequency somatic embryogenesis revealed alterations in both the nuclear and mitochondrial genomes [97]. These reports therefore proposed a critical evaluation of tissue culture-derived plants both at phenotypic and molecular level.

Adventitious shoot development is an alternative method of coffee micropropagation. Shoots originating in tissues located in areas other than leaf axils or shoot tips are subjected to one phase of dedifferentiation followed by differentiation and morphogenesis [68, 98]. Rooting is the most difficult and expensive phase of the micropropagation process, and the success of newly formed plantlets is closely linked to the ability of the root system to adapt to the autotrophic conditions. Several studies have been carried out to improve the rate of rooting of micropropagated plants [68].

## 6. Development of Transgenic Coffee

Genetic engineering research on coffee has been pursued for the past fifteen years with two major objectives: (1) to elucidate the function, regulation, and interaction of agronomically important genes through a functional genomics approach and (2) to improve coffee genotypes with desirable traits through the introduction of targeted genes.

### 6.1. Candidate Genes

In recent years, the development in high-throughput sequencing (HTS) technologies has allowed the rapid acquisition of significant amounts of sequence data, and this has increased our understanding of the genomics of a particular species. During the last decade, significant progress has been made in developing an EST database for coffee. Initial efforts in developing ESTs in *Coffea arabica* were initiated by the University of Trieste, Italy, and the EST sequences have been placed in the public domain (http://www.coffee.dna.net/). In a private/public collaboration between Nestle and Cornell University, 47000 ESTs from *C. canephora* were established comprising 13175 unigenes [63]. Subsequently the Brazilian government funded an ambitious coffee genome program, and this has resulted in the establishment of 200 000 ESTs which led to the identification of 30000 genes [64]. Very recently, the Italian group has generated an additional 161 660 ESTs which will be publicly available at the website (http://www.coffeedna.net/ [99]). In parallel with the development of EST database, BAC libraries of coffee species, *C. arabica* and *C. canephora* were established [65, 66]. Such maps are of central strategic importance for marker-assisted breeding, positional cloning of agronomical important genes, and analysis of gene structure and function.

Due to the concerted efforts on coffee genomics, many candidate genes from coffee have been identified and some of them have been cloned and are currently being characterised. These include a caffeine biosynthesis gene [28, 100], a sucrose synthase gene [66], osmotic stress response genes [101], genes for seeds oil content [102] and several pathogen resistance genes such as *Mex-1* gene [103], *SH *_3_ gene [104], and *Ck-1* gene to CBD [105]. An efficient genetic transformation protocol is necessary in order to validate the structural and functional aspects of these intrinsic genes. In coffee transformation experiments, genes isolated from coffee as well as from heterologous sources have been used. Some of the genes isolated from coffee used in transformation experiments include a theobromine synthase gene (*CaMXMTI*) for suppressing caffeine biosynthesis [28, 106] and an *ACC oxidase* gene involved in ethylene biosynthesis [34]. The genes introduced to coffee from heterologous sources include a *cry*1Ac gene from *Bacillus thuringiensis *targeted against leaf miner [26], the **α*-AII* gene from common bean for imparting resistance to coffee berry borer [30], the *BAR* gene for herbicide tolerance [21, 35], and the homeobox gene *WUSCHEL* (WUS) from *Arabidopsis* responsible for stem cell identity [36]. The functional significance and expression of these transgenes in coffee plants are described subsequently in this paper.

### 6.2. Promoters

In most of the coffee transformation experiments reported so far, with few exceptions, the CaMV35S promoter derived from cauliflower mosaic virus is extensively used in transgenic constructs (See Table 1). In a comparative study made earlier, the efficacy of different promoters driving *uidA* transient expression in endosperm, somatic embryos, and leaf explants of *C. arabica *was analyzed using microprojectile bombardment [18, 19]. It was observed that the EF-1*α* promoter (from *Arabidopsis thaliana* EF-1*α* translation elongation factor) directed maximum transient expression of the *uidA* gene compared to other promoters [18]. Therefore, this promoter was used subsequently by the same group in *Agrobacterium* gene constructs, driving the *cry1Ac* gene for the control of leaf miner in coffee [26, 33]. The efficacy of the CaMV35S viral promoter was also compared with two coffee promoters (*α*-tubulin and *α*-arabicin) which have revealed a similar level of transient *uidA* gene expression [19]. These findings have opened up the possibility of using coffee specific promoter in transformation experiments.

### 6.3. Reporter Genes

Reporter genes are used in gene constructs to optimize the transformation procedure. In the majority of coffee transformation experiments, the *uidA* gene is used as a reporter gene (See Table 1). Only recently have the *sgfp *(synthetic green fluorescence protein) and *DsRFP* (Red fluorescent protein) been used in coffee transformation [28, 31, 37]. However, most transient expression studies have been carried out using the *uidA *reporter gene. In an effort to optimize the *Agrobacterium* transformation protocol in coffee, the expression of the *uidA* gene driven by a CaMV35S promoter was compared in various tissues under different cocultivation conditions [27]. It was observed that the endogenous *GUS *activity was reduced substantially when 20% methanol was added to the *GUS* staining solution. Marked differences in *GUS* activity were observed between the endogenous, and transformed tissue as the transformed plants exhibited a deep blue colour in reaction with X-gluc, while nontransformed plants only exhibited a pale blue coloration. Several factors such as of cocultivation period, preculture of explants, and acetosyringone-influenced *GUS* activity. Furthermore, in a comparative study, it was observed that *GUS* expression in leaf explants was more pronounced in the cut ends of the veins, whereas zygotic and somatic embryos, hypocotyls, and the adjacent region are the main target sites [27]. Recently, in another experiment, the expression of p35S. GUS expression was found to be stronger in the root tip and central vascular system compared to other regions in the root system [43].

Recently, the expression of both *uidA *and *gfp *genes driven by the CaMV35S promoter was monitored using various *Agrobacterium* strains and culture conditions [37]. Expression of the *sgfp-S65T* gene driven by the CaMV35S promoter (signified by green fluorescence) was observed in cocultivated calli just 2 days after cocultivation. Initially, green fluorescence appeared as discrete spots but subsequently, calli that showed green fluorescence increased in size, producing a bright fluorescence mass after 15 days cocultivation. The expression of both *uidA *and *sgfp* was intense in globular- and torpedo-shaped embryos until the development of cotyledonary leaves. In older leaves, green florescence was weak due to the interference of chlorophyll, which emits red fluorescence at the same activating wavelength. The pattern of expression of *GUS* and *gfp *genes driven by the CaMV35S promoter was similar, being much more pronounced in the leaf veins and root tips and in vascular zones. Using the CaMV35S promoter, similar expression patterns were obtained for *DsRFP* in somatic embryos [31] and for* gfp* in roots [44].

## 7. Transformation Systems

Both direct and indirect DNA delivery systems have been employed to transform coffee by various workers and the details are described below.

### 7.1. Direct DNA Delivery

#### 7.1.1. Electroporation

Electroporation is a process through which permeability of the cell plasma membrane is significantly increased by the external application of electrical field. It is usually used in molecular biology as a way of introducing some substance into a cell, such as a drug that can change the cell's function, or a piece of coding DNA. Electroporation was used to integrate foreign DNA into protoplasts of *C. arabica* [15]. Regeneration of transformed embryos and plantlets resistant to kanamycin was obtained but the plantlets did not survive due to a weak root system. In another experiment, various parameters influencing transformation of coffee somatic embryos using electroporation of pCAMBIA 3201 plasmid carrying the *uidA* gene were described [16]. The results showed that the electroporation of somatic embryos at torpedo stage can be a promising approach to coffee transformation since transformed torpedo-shaped embryos produced significantly higher numbers of gus positive secondary embryos in the culture medium. Recently, the expression of *uidA* gene driven by the N-methyltransferase (NMT) promoter was studied by electroporation of coffee endosperm [17]. The results indicated that *uidA* gene expression driven by the NMT promoter is targeted to the external surface of the vacuoles.

### 7.2. Microprojectile Bombardment

Genetic transformation of coffee via microprojectile bombardment was described for the first time, using a gunpowder driven device and several target explants [18]. The study compared different promoters and demonstrated that the EF-1*α* promoter from *Arabidopsis thaliana* is more effective than the *CaMV35S* promoter in driving transient *GUS* expression in leaves of microcuttings. The interaction between osmotic preconditioning and physical parameters of helium gun device was studied in *C. arabica* suspension cells. It was observed that four hours of pretreatment of the target tissue with mannitol and sorbitol before bombardment increased the number of cells expressing *GUS* gene without causing cell necrosis [19].

Successful regeneration of transgenic coffee plants (*C. canephora*) by microprojectile bombardment was achieved by using the pCambia3301 plasmid containing the *uidA *and *bar* genes [21]. The study demonstrated the effectiveness of the *bar* gene for selection of transformants *in vitro* and *in vivo* identification of transgenic coffee plants. In *C. arabica*, the plasmid pBI-426 carrying the *nptII *and *uidA* genes was employed in particle bombardment of embryogenic calli, and transformants were selected using kanamycin [20]. The transgenic status of the regenerated plants was confirmed by PCR analysis. Recently, a *C. arabica* suspension culture with a high regenerative capacity for secondary somatic embryogenesis was used for transformation using microprojectile bombardment [22]. However, no transformants could be regenerated due to damage to bombarded tissue. Very recently, successful regeneration of transgenic *C. arabica* was reported using bombardment of embryogenic calli followed by kanamycin selection [23]. The authors reported the normal growth of the transgenic plants and obtained T_1_ progeny presenting 3 : 1 segregation of the *uidA* transgene.

### 7.3. Indirect DNA Delivery

*Agrobacterium tumefaciens. *The* Agrobacterium tumefaciens* mediated transformation technique has been extensively used for genetic transformation of coffee and is the method of choice for many workers (see Table 1). An initial report of *A. tumefaciens*-mediated transformation of coffee involved the cocultivation of protoplast with different *Agrobacterium* strains carrying *nptII *and *uidA* genes [24]. Transient GUS expression was demonstrated in the callus tissue derived from protoplasts but plant regeneration was not obtained. The first successful *A. tumefaciens* transformation and transgenic plant regeneration were achieved in *C. canephora *[25] and subsequently in *C. arabica* [26]. In *C. canephora,* various parameters that influence T-DNA delivery to coffee tissue were studied using transient GUS gene expression [27]. It was reported that preculture of explants prior to *Agrobacterium* cocultivation, addition of acetosyringone to cocultivation medium and the duration of the cocultivation period significantly influenced T-DNA delivery to coffee tissue. In addition to the culture conditions, the *A. tumefaciens* strains also influence the transformation efficiency in coffee [37]. *A. tumefaciens* strains and their plasmids are classified by the function of the opine genes they carry. These opines, which are synthesized in the infected plant cells, are mainly agropine, nopaline, and octopine. For coffee transformation, various *A. tumefaciens* strains such as LBA 4404 pAL4404 (octopine), C58CI pMP90 (nopaline), EHA 101 pEHA101 (agropine), EHA 105 pEHA101 (agropine), and AGL1 pTiBo542 (agropine) have been used by various workers (see Table 1). Recently, a study was carried out to compare the efficiency of four different *Agrobacterium* strains (LBA 4404, EHA101, EHA105, AGL1) in coffee transformation using pBECKS 2000 vector constructs carrying *uidA *and *gfp* reporter genes [37]. It was observed that EHA 105 and EHA101 were more efficient compared to LBA4404 in T-DNA delivery and transgenic plant regeneration. Based on this improved protocol, mass production of transgenic coffee plants of both *C. arabica* and *C. canephora* was achieved [37].

**Figure 1:**
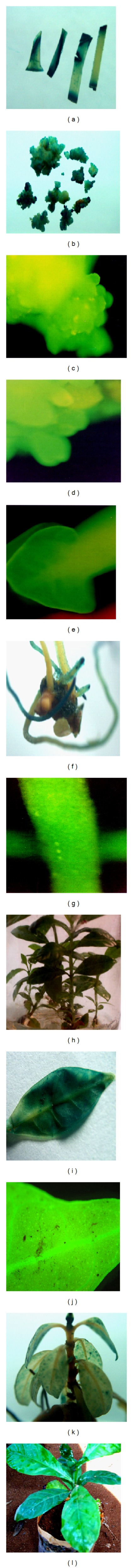
*Agrobacterium tumefaciens* mediated transformation and regeneration of *C. canephora *cv. C × R and *C. arabica* genotypes. (b, c, d, f, and k) *C. arabica* (a, e, g, h, i, j, and l) *C. canephora*. (a) Cocultivated hypocotyls of *in vitro* seedlings expressing transient GUS expression; (b) embryogenic calli showing transient GUS expression following cocultivation; (c) initiation of somatic embryos from the transformed calli showing green fluorescence; (d) mass of heart shaped somatic embryo showing green fluorescence; (e) germinating somatic embryo with well-developed cotyledon leaves with bright green fluorescence; (f) Gus expression in the root tips of a germinated transformed plant; (g) strong GFP expression in transgenic root; (h) *in vitro* plant regeneration; (i) Gus staining of the leaf of a transgenic plant; (j) GFP expression in the developing leaf; (k) GUS staining of the regenerated transformed plant; (l) transgenic plant in the soil.

In order to improve the efficiency of *A. tumefaciens* mediated transformation, sonication and vacuum infiltration methods were incorporated during cocultivation [31, 35]. In most of the *A. tumefaciens* mediated transformation, embryogenic tissues and/or somatic embryos were used as the target material for cocultivation (see Table 1). In *C. canephora,* a highly efficient *A. tumefaciens* transformation and regeneration protocol were established using hypocotyl explants as the target material (Figures 1(a)–1(l)) [29]. In *C. canephora*, the collar region of the hypocotyls was found to be more suitable for *A. tumefaciens* transformation [107]. However, in *C. arabica,* embryogenic calli were found to be more suitable for *A. tumefaciens* transformation [38]. The methodology for genetic transformation of *Coffea* using *Agrobacterium* was described in detail [108]. They were able to transform 20 different genotypes either belonging to *C. arabica* or *C. canephora* by cocultivation of embryogenic calli with *A. tumefaciens*.

*A. tumefaciens *mediated transformation has also been used for gene silencing using RNAi technology and several genes such as theobromine synthase (*CaMXMTI*) and N-methyltransferase (*NMT*) (both involved in caffeine biosynthesis) and the *ACC oxidase* gene (involved in ethylene biosynthesis) were targeted to coffee tissue in reverse orientation for obtaining stable silencing [28, 32, 34]. Recently, transgenic *C. canephora* plants incorporating a *homeobox* gene *WUSCHEL* (WUS) responsible for stem cell identity were regenerated [36].

*Agrobacterium rhizogenes. Agrobacterium rhizogenes* mediated transformation of both *C. canephora* and *C. arabica* species was reported as early as 1993 [39]. Subsequently, many workers have reported successful *A. rhizogenes* mediated transformation and plantlet regeneration in both *C. arabica* and *C. canephora* using different explants [40, 42, 44, 109]. In almost all cases, *Agrobacterium* strain A4 was used except in one case where bacterial strain IFO 14554 has been used (Table 1). Most of the binary constructs used in *A. rhizogenes* transformation are either based on a *pBIN19* or *pCambia* backbone. *A. rhizogenes* transformation is very useful for functional analysis of genes involved in resistance to root knot nematode in coffee.

## 8. Selection of Transformants

The successful recovery of stable transformants depends upon the choice of a suitable selective agent, its optimal concentration, timing, and frequency of selection. An effective selective agent must allow the growth and development of transformed tissue but simultaneously restrict the proliferation of nontransformed cells in the same culture medium. Therefore, incorporation of the right selectable marker gene in gene constructs is critical to the success of transformation. In coffee transformation, various selection marker genes (*hpt* hygromycin-R, *nptII* kanamycin-R, *csr1-I* chlor and sulfuron-R, *ppt* phosphinothricin-R) were used (Table 1), and their efficacy has been evaluated [18, 27]. The first work in coffee transformation was carried out using kanamycin as the selective agent [39]. However, contradictory results were obtained with regard to the efficacy of kanamycin for selection of coffee transformants. Many workers [15, 39, 41, 110] have reported the development of nontransformed somatic embryos at higher kanamycin concentrations and have attributed this to a poor capacity for transformant selection. However, there have also been several reports of successful selection of transformed coffee plants using kanamycin as the sole selective agent [20, 23, 31]. In many transformation experiments, hygromycin at concentrations of 20–100 mg/L was used successfully for selection of transformants [25, 27–29]. The efficiency of hygromycin as a selective agent was tested in different transformed and nontransformed tissue of *C. canephora* and it was observed that 25 mg/L hygromycin severely checked nontransformed somatic embryo growth and proliferation [27]. Similar results were also obtained by using hygromycin in *A. rhizogenes* mediated coffee transformation [42].

In addition to antibiotics, several other types of selection markers such as herbicide selection and positive selection have also been used in coffee transformation. The reliability of chlorsulfuron (*csr1-I*), phosphinothricin (*ppt*), and ammonium glufosinate (*bar*) as selection markers to regenerate transformed tissue were already confirmed in both *C. arabica* and *C. canephora* using various transformation methods [21, 26, 30]. As an alternative to negative markers such as antibiotics and herbicides, positive selection marker genes such as phosphomannose isomerase (*pmi*) and xylose isomerase (*xylA*) have also been used for coffee transformation, producing transformants able to grow in the presence of mannose and xylose, respectively, without an additional carbohydrate source. The study indicated that compared to mannose, xylose is an effective selective agent for coffee transformation [111].

In recent times, transgenic crops regenerated carrying antibiotic and herbicide resistance genes have generated public disquiet about food safety and environmental impact. This has stimulated research into utilizing visual selection markers instead of using antibiotic and/or herbicide selection markers. In coffee, green fluorescent protein, (*gfp*), and red fluorescent protein (*DsRFP*) were used for visual selection of transformed tissue following *A. tumefaciens* mediated transformation [28, 31, 37]. The regeneration of transformed roots of *C. arabica* using visual selection of green epifluorescence without using any selective agent was achieved through *A. rhizogenes* mediated transformation [44]. Recently transgenic plants of both *C. canephora* and *C. arabica* were regenerated by employing visual selection of green fluorescent protein as the sole screen following *A. tumefaciens* mediated transformation [112].

## 9. Transgene Expression

### 9.1. Expression of Reporter Genes

Studies pertaining to expression of transgenes in a perennial crop like coffee are very important. However, such reports are very limited. The expression of *uidA* and *gfp* transgenes driven by a CaMV35S viral promoter was monitored at different stages of plant growth of *C. canephora* following *A. tumefaciens* mediated transformation [27, 37]. It was observed that maximum expression of both *uidA* and *gfp* genes were obtained at the globular- and torpedo-shaped somatic embryos. Following the development of cotyledon leaves, GUS expression was scattered, with pronounced expression shifted to vascular regions in the well-developed leaves. In roots, maximum expression was obtained in the root tips and root hairs compared to the main roots. Recently, the expression of *uidA* gene driven by the double CaMV35S promoter was monitored in the flowers and fruits of *C. arabica *transgenic plants obtained through microprojectile bombardment [23].

### 9.2. Expression of Insect Resistance Genes

Transgenic coffee (*C. canephora*) plants incorporating synthetic *cry* genes (*cry1Ac*) from *Bacillus thuringiensis* were regenerated [26]. Field assessment demonstrated the resistance of transgenic plants to coffee leaf miner (*Perileucoptera coffeella*) indicating the functional stability of the transgenes [33]. In another study, transgenic plants of *C. canephora* incorporating *α*-A11 from common bean were produced via *A. tumefaciens* mediated transformation, and bioassays with the insect are underway to confirm functional validation of its protein in coffee [30].

### 9.3. Expression of Herbicide Resistance Genes

Transgenic coffee plants incorporating the *csr-1-1* gene were produced using *A. tumefaciens* mediated transformation [26]. In this study, plants were selected using chlorsulfuron but several nontransformed escapes were obtained, which suggests that the herbicide is not a very tight selective agent. In another study, transgenic *C. canephora* plants were produced using the *bar* gene [35]. Regenerated plants were sprayed with the herbicide ammonium glufosinate under green house conditions and showed no phytotoxicity effects.

### 9.4. Modification of Expression of Genes Controlling Biochemical and Physiological Traits

The expression of genes, involved in the caffeine biosynthesis pathway, was modified using RNAi technology to reduce the level of *CaMXMT1* (theobromine synthase) [28]. Transgenic plants expressing antisense *ACC oxidase* (involved in fruit maturation and ethylene production) have also been produced and stable expression of the transgene observed [34].

## 10. Applications of Transgenic Technology

Genetic transformation technology has potential applications in coffee agriculture by incorporating desirable traits such as disease and insect resistance, drought and frost, tolerance and herbicide resistance. Transgenic technology can also be used to increase nutritional value and improve cup quality, produce varieties with caffeine-free beans, and for production of hybrid crops for molecular farming. Identification of target specific genes is one of the pre-requisites for developing transgenic crops. The availability of a large number of EST sequences in coffee and initiation of coffee genome sequencing may speed up the gene discovery and accelerate transgenic research efforts in coffee.

### 10.1. Insect Resistance

Production of insect resistant coffee plants is one of the major objectives of the breeding programs. The major pests attacking coffee include coffee berry borer (CBB, *Hypothenemus hampei*), white stem borer (WSB, *Xylotrechus quadripes*), leaf miner (*Perileucoptera coffeela*), and root nematodes (*Meloidogyne* spp. and *Pratylenchus* spp.). The CBB is present in almost all the coffee growing countries and considered to the most devastating pest in coffee. To date, there is no reported source of resistance to CBB in the coffee gene pool. Like CBB, WSB is another serious pest in arabica coffee in India and several other East Asian countries. Both CBB and WSB belong to the order Coleoptera. For India, controlling WSB is the biggest challenge and has therefore become the highest research priority. Robusta is generally resistant to WSB but the interspecific robusta arabica hybrids are susceptible to WSB. Although leaf miner is not yet a serious pest in India and other East Asian countries, it is an economically important pest in East Africa and Brazil.

Effective chemical control of CBB and WSB is difficult due to the nature of their life cycle inside the berry and stem, respectively, as well as environmental concern regarding the use of pesticides. Biological control measures are adapted to combat these insect pests with varying degrees of success. Developing coffee plants resistant to these pests using genetic transformation technology could be one of the alternative strategies to counter pest damage.

For insect resistance, several different classes of proteins from bacterial, plant, and animal sources have been isolated and their insecticidal properties tested against many important pests. Amongst these proteins, Bt toxins are most important and several transgenic crops expressing Bt genes have been commercialized. Coffee transgenic plants carrying a synthetic version of the *cry1Ac* gene have been produced [26, 67]. Indeed, this was the first report that an important agronomic trait has been introduced into a coffee plant. The transgenic plants presented similar features in growth and development compared to normal plants. Transgenic plants highly resistant to leaf minor under greenhouse conditions were tested under field conditions in French Guyana for 4 years for field resistance [33, 113]. From a total of 54 independent transformation events, 70% of the events were resistant to leaf minor. Unfortunately, the field trial was vandalized which led to the termination of the experiment [114].

The effectiveness of *Bt* genes in controlling coleopteran pests is well documented in corn and potato, which indicates that Bt genes might be effective against CBB. The high toxicity of *B. thuringiensis *serovar* israelensis* against first instar larvae of CBB has been demonstrated [115]. In another experiment, an *α*-amylase inhibitor from *Phaseolus vulgaris* was tested against CBB and found to have an inhibitory effect on its growth and development [116].

In addition to the CBB and WSB, arabica coffee varieties are also susceptible to endoparasitic root-knot nematode (*Meloidogyne* spp.) [117]. So far, 15 species have been reported to be parasites of coffee. Controlling nematodes is extremely difficult and currently seedling grafting with robusta rootstock is followed as one of the control measure. Sources of resistance specific to root-knot nematodes have been identified in coffee trees [118] and the *Mex-1* gene conferring resistance to *M. exigua* in *C. arabica* is in the process of isolation [103]. The functional analysis of nematode resistant genes could be carried out by using the *A. rhizogenes* mediated transformation protocol already developed in coffee (See Table 1). Root-specific promoters could also be used in the vector constructs to drive transgene expression in the root.

Cultivation of insect resistant transgenic coffee should address ecological concerns related to insects and soil microorganisms. Transgene stability also needs to be studied since a coffee plantation can remain for several years without replantation. The constant selection pressure of the transgenic plants on the targeted insect population should be monitored, as there may be a chance of emergence of insect resistant populations.

### 10.2. Tolerance to Abiotic Stress

In many coffee growing countries, abiotic stress such as drought and frost are the major climatic factors that limit coffee production. Changes in climatic patterns due to global climate change is considered increasingly important for coffee cultivation. Drought induces water stress in plants, which affects vegetative growth and vigour, and triggers floral abnormalities and poor fruit set. It also indirectly increases the incidence of pests and diseases in the plants. Arabica is generally more tolerant to water stress than robusta, partly due to its extensive deep root system. *C. racemosa* is known to be a good source of drought tolerance. In India, hybrids between *C. canephora* and *C. racemosa* have been obtained and are currently under evaluation for drought tolerance.

In many coffee growing countries, coffee is propagated in marginal areas where the annual rainfall is below 1000 mm, with prolonged dry spells of over 4-5 months. In those areas, water shortage and unfavourable temperatures constitute major constraints, and the growth and productivity of robusta coffee are badly affected.

As with drought, periodic frost also affects coffee production in parts of Brazil. The introduction of drought and frost tolerant genes through genetic transformation would be of great importance for alleviating these problems. Research is now being carried out by several groups to identify genes involved in biotic as well as abiotic stress. The most promising approach of genetic engineering for drought tolerance includes the use of functional or regulatory genes as well as the transfer of transcription factors. In recent years, plants tolerant to high temperature and water stress have been the subject of intense research [119–121]. For achieving drought tolerance, genes that have been targeted include those encoding enzymes involved in detoxification or osmotic response metabolism, enzymes active in signalling, proteins involved in the transport of metabolites, and regulating the plant energy status [119–121]. The dissection of molecular mechanisms related to signal transduction and transcriptional regulation might help in engineering drought tolerance in coffee.

### 10.3. Disease Resistance

Coffee leaf rust (CLR) caused by the fungus *Hemileia vastatrix *is the most important disease in coffee with substantial loss to coffee production and productivity in all the coffee growing countries. In addition to leaf rust, coffee berry disease (CBD) caused by fungus *Colletotrichum kahawae* can be a devastating anthracnose causing substantial crop loss in Africa. Several other fungal and bacterial diseases may also affect coffee, causing economic damage to a small extent. Arabica is more susceptible to many diseases than robusta coffee. Though most of the disease control measures rely upon chemical control, they are more expensive and labour intensive. The long-term solution is the breeding of resistant varieties, which is the focus of many breeding programmes. However, breeding for disease resistant varieties is time consuming due to the perennial nature of coffee, with its long gestation period. India has a long history of arabica coffee breeding especially for leaf rust resistance and is the first country to demonstrate the existence of multiple races of leaf rust. Resistance to CLR is conditioned primarily by a number of major (*S*_*H*_) genes, and coffee genotypes are classified into different resistance groups based on their interaction with different rust races pathogen [122]. Currently *C. canephora* provides the main source of resistance to pests and diseases including CLR (*H. vastatrix*) and CBD (*C. kahawae*) and is therefore used in breeding programs. Other diploid species like *C. liberica* and *C. racemosa* have been used as a source of resistance to coffee leaf rust and coffee leaf miner, respectively [12, 123].

The development of coffee varieties resistant to major fungal diseases such as CLR and CBD using transgenic technology will benefit the coffee industry immensely. During the last 15 years, significant progress has been made in the area of host-pathogen interactions [124, 125] and many resistance genes involved in recognizing invading pathogens have been identified and cloned [126]. A number of signalling pathways, which are induced following pathogen infection, have been dissected [127]. Many antifungal compounds that are synthesized by plants to combat fungal infection have been identified [128]. Understanding the specific induction of targeted pathways and identification of specific pathways responsible for particular fungal resistance is important in order to employ this strategy in transgenic technology. The recent investigation of gene expression during coffee leaf rust infection could give an insight into the defence pathways operating in coffee [129, 130].

Efforts have been made to identify and clone resistance genes from coffee for achieving durable resistance. Recently, the genetic and physical map of two resistance genes, that is, the *S*_*H*_ 3 gene conferring resistance to rust [104] and the *Ck1* gene conferring resistance to *C. kahawae* CBD [105] have been established. These genes could be used for molecular marker assisted breeding programmes.

### 10.4. Production of Low-Caffeine Coffee

Low-caffeine and decaffeinated coffee represent around 10% of the coffee sales around the world [35]. The industrial process for coffee decaffeination can be expensive and affects the original flavour and aroma in coffee [131].

Transgenic coffee plants with suppressed caffeine synthesis using RNA interference (RNAi) technology have been obtained [28, 106]. Specific sequences in the 3′ untranslated region of the theobromine synthase gene (*CaMXMT1*) were selected for construction of RNAi short and long fragments. The caffeine and theobromine content of the transgenic plants reduced by up to 70% compared to the untransformed plants. In *C. canephora*, RNAi technology has also been employed to silence the N-methyl transferase gene involved in caffeine biosynthesis [17]. Recently the promoter of an N-methyltransferase (*NMT)* gene involved in caffeine biosynthesis was cloned [100], which will be very useful for studying the regulation of caffeine biosynthesis.

### 10.5. Improvement in Cup Quality

Improvement in coffee cup quality requires elaborate knowledge of the chemical constituents as well as the metabolic pathways involved in the elaboration of quality. The constituents of coffee beans include minerals, proteins, carbohydrates caffeine, chlorogenic acids (CGA), glycosides, lipids, and many volatile compounds that give flavour to coffee by roasting. Among these, the role of three major constituents: sucrose, CGA, and trigonelline have been studied in coffee. The sucrose content of coffee bean is associated with coffee flavour; the higher the sucrose content in green beans, the more intense will be the cup flavour [132, 133]. The sucrose content of *C. arabica* (8.2-8.3%) is higher than *C. canephora* (3.3–4.0%). The sucrose:amino acids ratio in green beans determines the profile of volatile compounds. Manipulating sucrose content in coffee bean is therefore important in improving cup quality. Recently, the sucrose synthase gene (*CcSUS2*) from *C. canephora* has been cloned and sequenced [66]. This provides an opportunity to manipulate the sucrose content in coffee.

Chlorogenic acids are products of phenylpropanoid metabolism. Chlorogenic acids are a group of hydroxycinnamoyl quinic acids (HQA) formed by esterification between caffeic acids, coumaric acids, and quinic acids [134]. They are present in relatively large quantities in the coffee bean and are the precursor of phenolic compounds in roasted beans. Robusta beans contain higher CGA (10%) compared to arabica beans (6-7%). CGA are known to have antioxidant properties as well as being associated with disease resistance [135]. Genetic manipulation of genes involved in CGA synthesis can serve either of these purposes by up- or downregulating the pathway.

Phenylalanine ammonia-lyase (PAL) catalyzes the first step of the phenylpropanoid pathway leading to the synthesis of a wide range of chemical compounds including flavonoids, coumarins, hydroxycinnamoyl esters, and lignins [136]. Recently, the full length cDNA and corresponding genomic sequences of PAL from *C. canephora* was isolated, characterized, and functionally validated [137, 138]. This has opened up new possibilities for manipulating the level of the PAL enzyme in coffee which in turn will be useful for cup quality improvement and manipulating antioxidant properties in coffee.

### 10.6. Fruit Ripening

Uniformity during fruit ripening is decisively related to cup quality in coffee, and consequently to the value of the product. Fruits at the ideal ripening stage produce the best organoleptic characteristics for coffee. The presence of overripened or green fruits changes the acidity, the bitterness and consequently the cup quality. In order to maximise uniform ripening of coffee fruits, it is essential to control the action of genes involved in the last step of maturation process. Ethylene is known to trigger ripening, and increasing ethylene biosynthesis is associated with various stages of ripening process [139]. To control coffee fruit maturation, two of the major genes involved in ethylene biosynthesis, namely, ACC synthase and ACC oxidase, have been cloned [139, 140]. Introduction of the *ACC oxidase *gene in antisense orientation has been achieved in both *C. arabica* and *C. canephora* [34]. The effect of the transgene on ethylene production and fruit maturation has yet to be reported. The inhibition of genes downstream to the initial ethylene burst is also an option to control coffee fruit maturation [141].

## 11. GM Coffee and Consumer Approval

Considering that the technology for coffee transformation is available, and given the rapid progress in gene discovery, it may not be very long before transgenic coffee hits the market. Since transgenic coffee is at the initial stages of commercial development and needs to be integrated into the main breeding programmes for evaluation, it will take at least 15–20 years for field release of GM coffee. However, the main obstacle will be consumer approval and acceptance of genetically modified coffee. With a rapid increase in the cultivation of various transgenic crops around the globe, consumer perception towards transgenic coffee may be more positive than it is today. Undoubtedly, GM coffee must undergo rigorous testing on both health and environmental effects before it is released for commercial cultivation.

## 12. Conclusion and Future Perspectives

During the last 15 years, transgenic crops became an integral part of the agricultural landscape. The number of transgenic crops and the area under cultivation is rapidly increasing in many parts of the world. This has been made possible by the application of genetic transformation technology and its integration with plant breeding programmes. Despite significant advances made over the last 15 years, coffee transformation is still time consuming and laborious. In addition, a genotype independent transformation protocol is not yet achieved in coffee. Genetic transformation of coffee has two major applications: (1) a tool for the validation of gene function and (2) production of transgenic crops with agronomically important traits. In order to achieve these goals, a simple, efficient, genotype-independent routine transformation protocol needs to be developed for coffee. Public concern regarding the use of antibiotic marker genes in transgenic technology need to be addressed. In this regard, coffee transformation should be based on several strategies such as use of positive selection markers and GFP for transformant selection, and on the *cre/lox *system for elimination of selectable marker genes. Development of a clean gene transgenic technology for coffee based on the *gfp* gene for visual selection and the *cre/lox* vector system is currently in progress. All coffee transformation programs should address the expression of transgenes in appropriate tissues, for which tissue specific promoters need to be used. The stable expression of transgenes should be monitored at every stage of plant growth and development. In addition, genomic technologies such as transgenics, molecular marker assisted breeding, genomics, proteomics, and metabolomics should complement traditional breeding efforts for hastening the genetic improvement of coffee.

Further coffee transformation programmes and investments should involve public and private companies. At the same time, researchers must make an effort to educate the public and help them understand the real advantages and risks associated with the use of genetically modified coffee. This is the only way to address irrational fears about the transgenic crops, and this will pave the way to the use of transgenic technology for coffee improvement.

## Figures and Tables

**Table 1:** Summary of transformation studies in *Coffea* sp^∗^.

DNA delivery method	Coffea species	Explants used	Binary vector	*Agrobacterium* strain	Promoter	Selection marker	Target gene	Results	Reference	Country
Direct delivery electroporation	*C. arabica*	Protoplast	NA	—	pGA472	*nptII*	*uidA*	TE	[15]	USA
	*C. arabica*	SE	pCAMBIA 3201	—	CaMV35S	*bar*	*uidA*	TGI	[16]	Venezuela
	*C. canephora*	Endosperm	pCAMBIA 1301	—	CaMV35S	*hpt*	*uidA*	TE	[17]	India

Microprojectile bombardment	*C. arabica*	Leaf	pPIGK	—	EF-1a	*bar*	*uidA*	TE	[18]	France
	*C. arabica*	ET	pCAMBIA 2301	—	CaMV35S	*nptII*	*uidA*	TGI	[19]	Colombia
	*C. arabica*	ET	pBI-426	—	CaMV35S	*nptII*	*uidA*	TGI, PR	[20]	Brazil
	*C. canephora*	ET	pCAMBIA 3301	—	CaMV35S	*bar*	*uidA*	TGI, PR	[21]	Brazil
	*C. arabica*	SE	pCAMBIA 2301	—	CaMV35S	*nptII*	*uidA*	TE	[22]	Costa Rica
	*C. arabica*	ET	pBI-426	—	CaMV35S	*nptII*	*uidA*	TGI, PR	[23]	Brazil

Indirect delivery *A. tumefaciens *	*C. arabica*	Protoplast	pGV2260	NA	CaMV35S	*hpt*	*uidA*	TE	[24]	France
	*C. canephora*	ET	pIGI121-Hm	EHA101		*hpt*	*uidA*	TGI, PR	[25]	Japan
	*C. arabica and C. canephora*	SE	pBIN19	LBA4404	EF-1*α*	*csr-1-1*	*cry1Ac*	TGI, PR	[26]	France
	*C. canephora*	ZE, SE, H	pBECKS 400	EHA101	CaMV35S	*hpt*	*uidA*	TGI,	[27]	India
	*C. arabica and C. canephora*	ET, SE	pHIBI –IG	EHA101	CaMV35S	*hpt*	*gfp & CaMXMTI*	RNAi, PR	[28]	Japan
	*C. canephora*	H	pBECKS 400	EHA 101	CaMV35S	*hpt*	*uidA*	TGI, PR	[29]	India
	*C. canephora*	ET	pCAMBIA 3301	EHA105	CaMV35S	*ppt*	*uidA*	TGI	[30]	Brazil
	*C. canephora*	ET, Leaf	pER10W-35SRed	C58	CaMV35S	*nptII*	*DsRFP*	TGI, PR	[31]	Mexico
	*C. canephora*	SE	pCAMBIA 1381	A4, EHA 101	CaMV35S	*hpt*	*NMT*	RNAi	[32]	India
	*C. canephora*	SE	pBIN19	LBA 4404	EF-1	*csr-1-1*	*cry1Ac*	Field test	[33]	France
	*C. arabica*	ET	pCAMBIA 3300	EHA 105	CaMV35S	*bar*	*ACC-oxidase*	AE	[34]	Brazil
	*C. canephora*	ET	pCAMBIA 3301	EHA 105	CaMV35S	*bar*	*uidA*	TGI, PR	[35]	Brazil
	*C. canephora*	Embryos	pER10W-35SRed	C58C1	CaMV35S	*WUSCHEL*	*DsRFP*	TGI,PR	[36]	Mexico
	*C. canephora and C. arabica*	ET	pBECKS 2000	EHA 101, EHA 105, LBA 4404, AGL 1	CaMV35S	*hpt, visual*	*uidA, sgfp*	TGI, PR	[37]	India
	*C. arabica*	ET	pMDC32	LBA1119	CaMV35S	*hpt*	*sgfp*	PR	[38]	France

*A. rhizozenes*	*C. arabica and C. canephora*	SE	pBIN 19	A4	CaMV35S	*hpt*	*uidA*	TGI, PR	[39]	France
	*C. arabica*	Leaf	NA	IFO 14554		NA	NA	TGI, PR	[40]	Japan
	*C. canephora*	SE	pBIN 19	A4	NA	*csr-1-1*	*cry1Ac*	TGI, PR	[41]	France
	*C. canephora*	SE	pCAMBIA 1301	A4	CaMV35S	*hpt*	*uidA*	TGI, PR	[42]	India
	*C. arabica*	H	pBIN 19	A4	CaMV35S	visual	*uidA*	TGI, PR	[43]	France
	*C. arabica*	H	pCAMBIA 2300	A4	CaMV35S	visual	*gfp*	TGI, PR	[44]	France
					CaMV35S					

^∗^Updated from Etienne et al. [45]; ET: embryogenic tissue; SE: somatic embryos; H: hypocotyls; TE: transient gene expression; TGI: target gene integration; PR: plant rregeneration; RNAi: RNA interference, AE: antisense expression.
